# On–Off Scheduling for Electric Vehicle Charging in Two-Links Charging Stations Using Binary Optimization Approaches

**DOI:** 10.3390/s21217149

**Published:** 2021-10-28

**Authors:** Rafał Zdunek, Andrzej Grobelny, Jerzy Witkowski, Radosław Igor Gnot

**Affiliations:** 1 Faculty of Electronics, Photonics and Microsystems, Wroclaw University of Science and Technology, Wybrzeze Wyspianskiego 27, 50-370 Wroclaw, Poland; andrzej.grobelny@pwr.edu.pl (A.G.); jerzy.witkowski@pwr.edu.pl (J.W.); 2 Elocity Sp. z o.o., pl. Wolnica 13/10, 31-060 Krakow, Poland; igor@elo.city

**Keywords:** electrical vehicles, EV charging scheduling, binary linear programming, binary quadratic programming

## Abstract

In this study, we deal with the problem of scheduling charging periods of electrical vehicles (EVs) to satisfy the users’ demands for energy consumption as well as to optimally utilize the available power. We assume three-phase EV charging stations, each equipped with two charging ports (links) that can serve up to two EVs in the scheduling period but not simultaneously. Considering such a specification, we propose an on–off scheduling scheme wherein control over an energy flow is achieved by flexibly switching the ports in each station on and off in a manner such as to satisfy the energy demand of each EV, flatten the high energy-consuming load on the whole farm, and to minimize the number of switching operations. To satisfy these needs, the on–off scheduling scheme is formulated in terms of a binary linear programming problem, which is then extended to a quadratic version to incorporate the smoothness constraints. Various algorithmic approaches are used for solving a binary quadratic programming problem, including the Frank–Wolfe algorithm and successive linear approximations. The numerical simulations demonstrate that the latter is scalable, efficient, and flexible in a charging procedure, and it shaves the load peak while maintaining smooth charging profiles.

## 1. Introduction

The electrification of transportation offers multiple benefits, including the reduction of noise pollution, fumes, and GHG emissions [1,2]. Hence, it has attracted increasing attention in recent decades, both from industry and academia, leading to considerable growth in numerous electric vehicles (EVs) in several countries globally. Consequently, there is a great need to develop an EV charging station infrastructure while maintaining a balanced load of power supply lines [3]. To pursue this goal, EV charging technology must be developed not only for fast charging systems but also for balanced overnight charging strategies with smart management of accessible energy, user energy demands, and cost savings. One of these strategies can be adopted in distributed farms of slow EV charging stations located in residential areas. Such farms are usually established in residential parking lots and are equipped with Level-II AC charging stations, controlled by the centralized coordinated scheduling unit.

In this study, we tackle the problem of smart coordination of the EV charging process in such farms, considering power supply constraints, balanced and smooth load expectations, energy demands, and user transportation habits and needs. The proposed strategies for controlling the EV charging process are mainly addressed for overnight charging, wherein the EVs to be charged and the transportation habits of their owners are usually known to the charging management system. However, vehicles do not have to be parked in the designed spaces. For such EVs, the expected daily energy demands can be easily estimated based on the charging history and parameters of the EV battery. Otherwise, new users or users who wish to modify their statistical daily charging demands should introduce updated data to the system before the initialization of the charging process. The system estimates the expected energy demand for each EV. However, the procedure for pursuing this task is not discussed in this paper.

The charging stations are currently categorized as Levels I, II, and III, depending on the range of power, which considerably affect the charging time. Level-I charging units usually operate on an AC electric power of 120 V and yield a power of approximately 1.4 kW. Level-II chargers offer power ranging from 4.4 kW to 22 kW and operate at 230 V AC. Direct current fast chargers, sometimes referred to as Level-III chargers, can charge with a maximum output of up to 350 kW, and it takes approximately 20–40 min to fill an EV battery to approximately 80% of its capacity.

**Figure 1:**
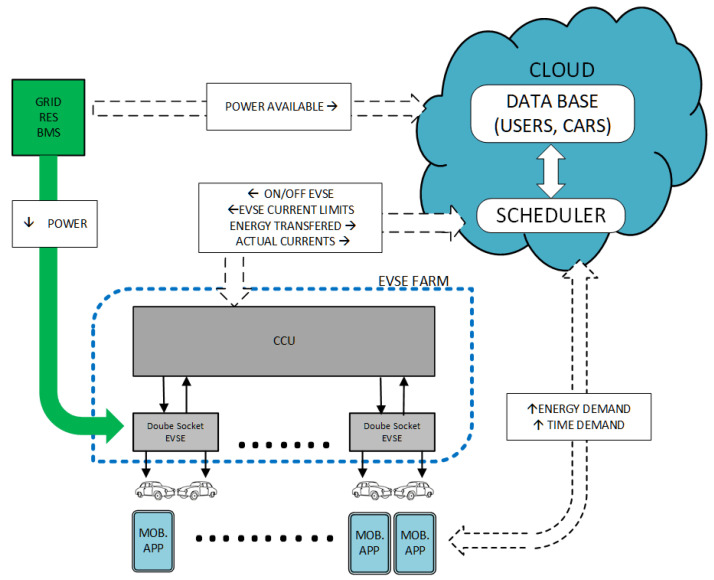
Block diagram of the analyzed farm of EV charging stations.

We assume that the analyzed farm contains three-phase Level-II AC charging stations, each equipped with two charging ports that can be controlled by the central control unit (CCU) and can serve up to two EVs but not simultaneously. The three-phase charging currents are measured at each station, and this information is back-forwarded to the CCU and then to the scheduler located on the cloud platform. The block diagram of the analyzed system architecture of the EV charging stations is shown in Figure 1. The charging stations (referred to as double-socket EVSEs) communicate with the scheduler via the CCU. The user–cloud interface is served by a mobile application that allows users to input its energy and time demand to the system and be informed by the status of its EVs’ charging.

The scheduler aims to determine an optimal scheduling for a charging process in the farm based on available data (both from the CCU and the database) and to accordingly control the charging stations. In this study, we assume that only a binary control over the charging station is possible, i.e., the charging link between the charging station and the connected EV can be activated or deactivated in a given time slot. This type of scheduling is referred to as on–off scheduling. The period between the plug-in and plug-off time was discretized, thereby allowing us to control the EV charging process using binary variables. The farm is supplied with three-phase power supply lines, and the scheduling should be determined in such a manner that the current consumption of the whole farm from each phase line does not exceed the assumed limits at any time. Moreover, the designed system should ensure a balanced power load over the entire accessible period. Performing this task is difficult because we expect cumulations for energy demands. Many users may plug-in their EVs in a relatively narrow period, possibly leading to a peak of high energy-consuming load if no control over the charging process is applied. The objective of the proposed scheduling scheme is to shave the load peak and ensure that the energy load is approximately constant throughout the entire accessible period. In our approach, we also assume that there is feedback between any working charging station and the scheduler, and the information on the charging currents is back-forwarded to the cloud in the discrete time, for example, every few minutes. The scheduler can update the current binary variables if any significant change in the charging currents is observed, or new EVs are recognized by the system.

Considering the above-mentioned physical limitations and user preferences, we propose three models for scheduling the EV charging process. The models are expressed in terms of numerical optimization problems with binary variables. In the first approach, we assume that the objective function is linear and the weighting coefficients are selected in such a manner as to execute the first-come first-served rule, subject to equality and inequality constraints. The equality constraints express the energy demands, whereas the inequality constraints refer to the power and available time limitations. Next, we extend the objective with a quadratic term that aims to enforce local smoothness to minimize the total number of switching operations. Frequent on/off switching of the battery might lead to a slightly lower battery life expectancy and to an increased number of transient instability effects (voltage ripples, overshooting, current peaks, among others). The quadratic model provides more flexibility in controlling the load profile. In the second approach, the objective function is formulated to maximize the constrained total energy consumption in each time slot, increasing the possibility of fast charging. In the last scenario, the optimization is triggered to prioritize the expected load demand for each EV. The last two models were expressed in terms of binary quadratic programming problems.

A binary linear programming (BLP) problem is not difficult to solve and can be regarded as a particular case of more general integer linear programming. Such problems are usually solved using relaxation or bound methods, including cutting planes, branch-and-bound, branch-and-cut, and many heuristics. In our approach, we need to solve a binary quadratic programming (BQP) problem, which is more challenging than a BLP problem. To address this, we analyze three computational algorithms—Frank–Wolfe (FW) [4], successive linear approximations (SLA) [5], and SLA with gradient descent updates. In the first case, the gradient of the objective function was minimized. It is a linear term for a quadratic objective function that can be easily addressed with the BLP. In the other cases, a BQP problem is reformulated to a BLP subject to quadratic inequality constraints that are subsequently linearized using SLA.

The remainder of this paper is organized as follows. Section 2 reviews related studies on scheduling schemes for EV charging. The proposed models for two-link charging stations and related computational strategies are presented in Section 3. Numerical experiments performed on various EV charging scenarios are presented and discussed in Section 4. The final section provides concluding statements.

## 2. Related Works

The optimal scheduling of EV charging has been a challenging problem and has been extensively studied in the last decade. There are many approaches and computational strategies for this problem, which are conditioned with a variety of charging station configurations, characteristics, and coordination objectives. A survey of recent trends in EV charging strategies and related optimization techniques can be observed in [6,7,8,9].

One of the main strategies, known as a decentralized or distributed strategy for EV charging [10,11,12,13,14,15,16], assumes that a major scheduling problem is decomposed into local subproblems individually tackled by charging stations based on the central reference information such as the electricity price or a reference power load. This type of coordination is much more flexible for users and computationally more efficient; however, this system is more prone to a load peak effect, possibly resulting in an overload of power grid components. The suppression of the load peak effect is one of the most important tasks in EV charging management, and it has been extensively discussed in the literature [17,18,19,20,21].

In another approach, the centralized management of a charging process constitutes a fundamental concept. Many studies [22,23,24,25,26] have highlighted the advantages of this type of charging strategy. In this approach, an EV charging control decision is taken globally by an aggregator determining the charging schedule and the rate. Central resource management is much more efficient in the optimization of power consumption and user demands. This strategy requires efficient real-time bidirectional communication between an aggregator, a CCU, and charging stations. It also involves a higher computational cost than a decentralized version; hence, it requires the use of fast scheduling algorithms.

Another issue related to the efficient management of an EV charging process is the selection of objectives and control types. The former may be formulated as an objective function that should be minimized, subject to various constraints. This might be due to the minimization of charging costs [27,28], tardiness [18], power imbalance [29], energy loss [30], grid loss [31], among others. The latter refers to the following options: spatial management (which EVs to charge in a given time instant), time management (when to switch on/off the stations), and available power management (how to determine the charging rate). The latter offers higher flexibility and can be combined with other options. Many studies [22,24,32] refer to a continuous charging rate control; however, this approach, despite its easy algorithmic feasibility owing to continuous variable optimization, is difficult to accomplish in practice. Discrete control over charging currents is easier to perform using digital electronic circuits governed by microcontroller units. Examples of papers discussing scheduling systems with a discrete charging rate can be obtained in [11,33].

A particular case of discrete charging rate control is a binary approach to the charging process. Several studies have demonstrated the efficiency of various on–off scheduling strategies in the context of optimal EV charging. Baek et al. [34] proposed a queuing model with random interruptions of charging EVs to relax the overload problem. Harris et al. [35] used a probabilistic approach for EV chargers modulating on/off, while Nguyen et al. [36] performed a similar task using the BLP combined with the bisection scheme. On–off scheduling schemes have also been studied by Fernandes et al. [37], who developed a dynamic charging scheduler based on greedy and LP algorithms. Another binary scheduling scheme was discussed in [38], where an EV-coordinated discrete charging problem was formulated in terms of a BQP problem and solved using an alternating switching algorithm. Recently, Jawad et al. [39] proposed a real-time EV charge management system that is based on a convex relaxation of on–off scheduling. In this approach, the binary constraint is relaxed to solve the on–off scheduling problem as a convex problem using LP, and then, a modified mapping is used to convert the solution back to binary values. However, this strategy cannot be adapted to the two-link charging stations in our farm because the two-link charging constraint in our model cannot be relaxed in this way.

Binary scheduling may also have some disadvantages. It is thus well known that a large number of charging cycles decreases battery life, as reported in [40,41,42]; however, Vroey et al. [43] argued that this degradation is marginal. Moreover, there is no evidence that switching between a minimal charging rate and its largest possible value affects the battery life.

In centralized charging systems, fast scheduling is a challenging problem that can be tackled using various computational strategies. Continuous variable optimization methods are usually used, such as LP [22,24,32,44,45] and QP [10,12,17,46]. A discrete charging rate control or any coordination scheme with integer decision variables requires the formulation of a non-convex optimization problem or sometimes even an NP-hard problem [47]. Therefore, integer-variable optimization tools must be used. Examples include BLP [37], BQP [38], MILP [48,49,50] and various metaheuristics with integer variables, including genetic algorithms [51], ant colony optimization (ASO) [13], particle swarm optimization [52,53], tabu search [54], memetic algorithm [55], artificial bee colony algorithm [56], binary evolutionary programming [57], and other greedy algorithms [33,58]. Other approaches to scheduling can also be obtained in the literature, for example, agent-based scheduling [59,60], deep reinforcement learning [61], and deep learning [62].

Motivated by the advantages of centralized EV charging strategies, we propose a new model and algorithmic approach for scheduling EV charging in such a strategy. The configuration of our charging station is similar to that presented in [56], that is, each charging station has two Type-II charging points, but each EV can be charged from a three-phase power line, and we have only one master or CCU that controls all charging stations in one farm. Motivated by the on–off scheduling in [37], we formulate a similar model with binary decision variables, but our algorithmic approach is completely different. Our algorithmic approach is motivated by the SOCDC algorithm given in [38], but the smoothing is enforced by a regularization term instead of the alternating optimization. We use different numerical approaches to solve QP problems; our model is also different, containing additional physical constraints for two-link charging stations, and our objective function is formulated such as to prioritize user demands.

## 3. Scheduling Problem

Notations:Boldface uppercase letters (e.g., ***X***) denote matrices; lowercase boldface letters represent vectors (e.g., ***x***); non-bold letters (e.g., *x*) are scalars; calligraphic uppercase letters (e.g., X) will be used to denote sets. The *j*-th column of ***X*** is denoted by $x_{j}$, and ${\underline{x}}_{j}$ is the *j*-th row vector of ***X***. The symbol $\left|\right|\cdot {\left|\right|}_{F}$ denotes the Frobenius norm of a matrix; ||·|| denotes the 2-nd norm. The sets of real numbers, natural, and binary (0–1) numbers are represented by R, N, and B, respectively. The symbols ⌊x⌋ and ⌈x⌉ denote the floor and ceiling functions of *x*, respectively.

### 3.1. Problem Formulation

In this section, we formulate a static scheduling problem in the form of a mathematical model with discrete time. The control horizon is expressed in terms of available time, which is partitioned into *T* time slots of equal length, for example, a few minutes. We assume that the farm contains *M* charging stations that can serve *N* EVs. As each station is equipped with two charging points (ports), we have N=2M. Let T={1,…,T} and N={1,…,N} contain the indices of time slots and charging points, respectively. The charging points are subsequently indexed, and *N* charging points can serve up to *N* EVs. The plug-in and plug-off times of the *n*-th EV are denoted by $t_{n}^{\left(in\right)}$ and $t_{n}^{\left(off\right)}$, respectively. Obviously, $1\leq t_{n}^{\left(in\right)}<t_{n}^{\left(off\right)}\leq T$. Without loss of generality, we assume that the first and second EVs are assigned to the first charging station, and the *n*-th and (n+1)-th EVs are assigned to the *m*-th charging station, where $m=\lfloor \frac{n+1}{2}\rfloor$ for n=1,…,N−1. Let $B=\left[b_{tn}\right]\in B^{T\times N}$ be a binary matrix indicating the available time for each EV, where

(1) $$\begin{array}{c} b_{tn}=\left\{\begin{array}{cc} 1 & \mathrm{if}n\text{-}\mathrm{th}\mathrm{EV}\mathrm{is}\mathrm{available}\mathrm{in}t\text{-}\mathrm{th}\mathrm{time}\mathrm{slot},\\ 0 & \mathrm{otherwise} \end{array}\right. \end{array}$$

The available time of the *n*-th EV can be computed as $T_{n}=\sum_{t=1}^{T}b_{tn}$, and it represents the number of time slots between $t_{n}^{\left(in\right)}$ and $t_{n}^{\left(off\right)}$. If $T_{n}=0$ for any n∈N, then there is no EV in the *n*-th charging point.

Let $c_{n}\leq T_{n}$ denote the energy demand of the *n*-th EV, which is the number of time slots required to charge a given EV to its desired state of charge (SoS) level. The charging rate for the *n*-th EV is represented by the parameter $r_{n}^{\left(l\right)}$, where l∈{1,2,3} represents the index of the line in the Type-II three-phase charging cable. In our approach, the changing rate refers to a quasi-stationary current (amperes/time slot) and does not have one constant value, but it is individually set for each EV and for each phase. This setting is motivated by the assumption that the schedule can be adjusted during charging considering the feedback information from charging stations, rates are time-dependent, and EVs can be scheduled in different periods of the available time.

Considering the power supply limit, let $I_{t}^{\left(max,l\right)}$ be the maximal *l*-th line current supplying the entire farm in the *t*-th time slot. The scheduler should assure possible fast charging but simultaneously not exceed $I_{t}^{\left(max,l\right)}$ for any time instant and phase. A separate current limit for each line is also required to avoid power imbalance across phases when the lines are unequally loaded.

To control the charging process, that is, which and when charging points to switch on/off, we introduce binary decision variables that form the scheduling matrix $U=\left[u_{nt}\right]\in B^{N\times T}$. The decision variables are defined as follows:

(2) $$\begin{array}{c} u_{nt}=\left\{\begin{array}{cc} 1 & \mathrm{if}n\text{-}\mathrm{th}\mathrm{EV}\mathrm{is}\mathrm{charging}\mathrm{in}t\text{-}\mathrm{th}\mathrm{time}\mathrm{slot},\\ 0 & \mathrm{otherwise}. \end{array}\right. \end{array}$$

The objective of the scheduler is to estimate the matrix ***U*** based on given criteria and available data, such as available time matrix ***B***, energy demand $\left\{c_{n}\right\}$, charging rates $\left\{r_{n}^{\left(l\right)}\right\}$, and maximal line currents $\left\{I_{t}^{\left(max,l\right)}\right\}$.

A fundamental requirement from the users is to charge their EVs to satisfy the given SoC level, and considering the variables defined above, this requirement can be determined by the following model:

(3) $$\begin{array}{c} \sum_{t\in [t_{n}^{\left(in\right)},\;t_{n}^{\left(off\right)}]}u_{nt}=c_{n},\;\forall n\in N, \end{array}$$

which can be equivalently expressed in the matrix equality constraint:

(4) $$\begin{array}{c} \mathrm{diag}\{UB\}=c, \end{array}$$

where $c={\left[c_{1},\ldots ,c_{N}\right]}^{T}\in N^{N}$ is the vector of the energy demands. Equation (4) restricts the feasible region to the periods determined by the plug-in and plug-off times. As all entries outside the periods are equal to zero, the energy demands can be reinforced by an additional equality constraint:

(5) $$\begin{array}{c} Ue_{T}=c, \end{array}$$

where $e_{T}={\left[1,\ldots ,1\right]}^{T}\in R^{T}$.

The farm of chargers is supplied with three-phase power with distribution transformers or other suppliers that have limited power. Thus, we assume that the maximal line current $I_{t}^{\left(max,l\right)}$ is not exceeded in any time slot, and this requirement can be modeled as follows:

(6) $$\begin{array}{c} \sum_{n\in N}r_{n}^{\left(l\right)}u_{nt}+\xi_{t}^{\left(l\right)}\leq I_{t}^{\left(max,l\right)},\;\forall t\in T,\;\mathrm{and}\;l\in \left\{1,2,3\right\}, \end{array}$$

where $\xi_{t}^{\left(l\right)}$ is the basic current obtained from the *l*-th line in the *t*-th time slot, which is the difference between the cumulative charging phase current and the line current from the transformer. The model (6) can be rewritten in the following matrix form:

(7) $$\begin{array}{c} e_{N}^{T}\left(D_{R}^{\left(l\right)}U\right)+{\underline{\xi }}^{\left(l\right)}\leq {\underline{I}}^{\left(max,l\right)},\;\mathrm{for}\;l\in \left\{1,2,3\right\}, \end{array}$$

where $e_{N}={\left[1,\ldots ,1\right]}^{T}\in R^{N}$, $D_{R}^{\left(l\right)}=\mathrm{diag}\left\{r_{n}^{\left(l\right)}\right\}\in R^{N\times N}$, ${\underline{\xi }}^{\left(l\right)}=\left[\xi_{1}^{\left(l\right)},\ldots ,\xi_{T}^{\left(l\right)}\right]\in R^{1\times T}$ and ${\underline{I}}^{\left(max,l\right)}=\left[I_{1}^{\left(max,l\right)},\ldots ,I_{T}^{\left(max,l\right)}\right]\in R^{1\times T}$.

In our configuration, each charging station has two charging points, which means that two EVs can be connected to one station, but the electronic instrumentation inside the station allows us to charge only one EV at any time instant. Switching between charging points is possible at any time and should be controlled by the CCU. Mathematically, this feature can be considered in a scheduling problem by formulating an inequality constraint that does not allow the setting of more than one decision variable to one for each charging station in any time slot. To tackle this problem, we define the two-link constraint matrix as

(8) $$\begin{array}{c} S=\left[\begin{array}{ccccccc} 1 & 1 & 0 & 0 & \cdots & 0 & 0\\ 0 & 0 & 1 & 1 & \cdots & 0 & 0\\ \vdots & \vdots & \vdots & \vdots & \ddots & \vdots & \vdots \\ 0 & 0 & 0 & 0 & \cdots & 1 & 1 \end{array}\right]\in B^{N/2\times N}. \end{array}$$

Subsequently, the two-link charging constraint can be formulated as follows:

(9) $$\begin{array}{c} SU\leq E_{N/2\times T}, \end{array}$$

where $E_{N/2\times T}=\left[\begin{array}{ccc} 1 & \cdots & 1\\ \vdots & \ddots & \vdots \\ 1 & \cdots & 1 \end{array}\right]\in B^{N/2\times T}$ is a matrix of all ones.

Considering the constraints in (4), (5), (7), and (9), the scheduling problem can be formulated as the following constrained optimization problem:

(10) $$\begin{array}{ccc} & & \min_{U\in B^{N\times T}}\Psi \left(U\right),\\ & s.t. & \;\mathrm{diag}\{UB\}=c,\\ & & Ue_{T}=c,\\ & & e_{N}^{T}\left(D_{R}^{\left(l\right)}U\right)+{\underline{\xi }}^{\left(l\right)}\leq {\underline{I}}^{\left(max,l\right)},\;\mathrm{for}\;l\in \left\{1,2,3\right\},\\ & & SU\leq E_{N/2\times T}, \end{array}$$

where Ψ(U) is an objective function.

We studied various approaches to define the objective function, which can be stated as follows:

- *Linear:* Motivated by the concept of the objective weighting, given in [38], we formulate the weighted linear function:
  (11) $$\begin{array}{c} \Psi \left(U\right)=e_{N}^{T}Uw, \end{array}$$
  where $w={\left[e_{t_{w}+1}^{T},\vartheta +1,2\vartheta +1,\ldots ,\vartheta \left(T-t_{w}\right)+1\right]}^{T}\in R^{T}$, where $\vartheta =\frac{w_{max}-1}{T-t_{w}}$. The parameters $t_{w}$ and $w_{max}$ were experimentally set to $t_{w}=\left\lfloor \frac{T}{10}\right\rfloor$ and $w_{max}=10$. Vector ***w*** represents a piecewise linear function. The first $t_{w}$ entries are equal to one, whereas the others linearly increase from one to $w_{max}$. Such weighting aims to penalize decision variables in later time slots, which should enforce charging as early as possible. Minimization of a linear function can be performed using any BLP solver. This approach is computationally efficient; however, it is not flexible owing to the limited possibility of using multiple penalties.
- *Quadratic I:* In this model, we assume charging of all EVs with a possibly maximum power, which leads to the following objective function:
  (12) $$\begin{array}{c} \Psi \left(U\right)=\frac{1}{2}\sum_{l=1}^{3}{∥{\underline{I}}^{\left(max,l\right)}-{\left(r^{\left(l\right)}\right)}^{T}UD_{w}∥}_{2}^{2}, \end{array}$$
  where $r^{\left(l\right)}=\left[r_{n}^{\left(l\right)}\right]\in R^{N}$ for l∈{1,2,3}, and $D_{w}=\mathrm{diag}\left\{w\right\}\in R^{T\times T}$. Matrix $D_{w}$ has a task similar to that of the linear function.
- *Quadratic II:* Another possibility is to reinforce the SoC level bilanse with additional weighting of time slots. This task can be achieved using the following objective function:
  (13) $$\begin{array}{c} \Psi \left(U\right)=\frac{1}{2}{∥c-UD_{w}e_{T}∥}_{2}^{2}. \end{array}$$
- *Penalized quadratic with smoothness constraints:* None of the above-mentioned objective functions assures a smooth solution, indicating that the number of switching on/off charging stations is not controlled within the area of feasibility bounded by the constraints. However, the number of switching operations can be minimized by introducing a trade-off between the model fitting and the local smoothness measure. Taking into account the objective functions (12) and (13), the degradation of model fitting by adding a regularization or penalty term is not a problematic issue because the model constraints are explicitly added to the optimization problem and guarantee feasibility.
  The local smoothness of the charging profile for each EV can be measured according to the following function:
  (14) $$\begin{array}{c} \Phi \left(U\right)=\frac{1}{2}\sum_{n\in N}\sum_{t=1}^{T-1}{\left(u_{n,t}-u_{n,t+1}\right)}^{2}. \end{array}$$
  Let ***L*** be the first-order differential operator defined as:
  (15) $$\begin{array}{c} L=\left[\begin{array}{cccc} 1 & -1 & 0 & 0\\ 0 & \ddots & \ddots & 0\\ \ddots & \ddots & \ddots & -1\\ 0 & \ddots & 0 & 1. \end{array}\right]\in R^{T\times T} \end{array}$$
  The function Φ can be equivalently rewritten using matrix ***L*** in the form $\Phi \left(U\right)=\frac{1}{2}{∥UL^{T}∥}_{F}^{2}$. Consequently, the objective function (13) with the additive smoothness penalty term is given by
  (16) $$\begin{array}{c} \Psi \left(U\right)=\frac{1}{2}{∥c-UD_{w}e_{T}∥}_{2}^{2}+\frac{\alpha }{2}{∥UL^{T}∥}_{F}^{2}, \end{array}$$
  where α≥0 is a penalty term.

For the above objective functions, the scheduling problem in (10) can be regarded as a BLP problem with the objective function (11) or the constrained BQP problem with the other functions. Regardless of the objective function, the problem expressed in the form (10) cannot be directly solved with standard numerical optimization solvers because the solution has the form of a matrix that cannot be successively processed with respect to its rows or columns. This results from the column and row action constraints that must be satisfied simultaneously. However, simple vectorization operations can be applied to transform the existing matrix equations into their equivalent vector forms.

**Lemma** **1.** *Formula (4) can be equivalently expressed in the form:*

(17) $$\begin{array}{c} {\left(B⊙I_{N}\right)}^{T}u=c, \end{array}$$

*where $u=\mathrm{vec}\left(U\right)\in B^{NT}$ is a vectorized version of the matrix **U**, and the symbol ⊙ denotes the Khatri–Rao product.*

The proof of Lemma 1 is given in Appendix A.

**Definition** **1.** *Let $A\in R^{M\times I}$, $X\in R^{I\times J}$, and $Y\in R^{J\times N}$. Then:*

(18) $$\begin{array}{c} \mathrm{vec}\left(AXY\right)=\left(Y^{T}⊗A\right)\mathrm{vec}\left(X\right). \end{array}$$

Applying Formula (18) to (5), where $A=I_{N}$, X=U, and $Y=e_{T}$, we obtain:

(19) $$\begin{array}{c} (e_{T}^{T}⊗I_{N})u=c. \end{array}$$

Similarly, the inequalities (7) and (9) can be reformulated using Formula (18) as follows:

(20) $$\begin{array}{c} \left(I_{T}⊗{\left(r^{\left(l\right)}\right)}^{T}\right)u\leq {\tilde{I}}^{\left(max,l\right)},\;\mathrm{for}\;l\in \left\{1,2,3\right\}, \end{array}$$

(21) $$\begin{array}{c} \left(I_{T}⊗S\right)u=\left(I_{T}⊗I_{N/2}⊗e_{2}^{T}\right)u=\left(I_{NT/2}⊗e_{2}^{T}\right)u\leq \hat{e}, \end{array}$$

where ${\tilde{I}}^{\left(max,l\right)}={\left({\underline{I}}^{\left(max,l\right)}-{\underline{\xi }}^{\left(l\right)}\right)}^{T}$ and $\hat{e}=\mathrm{vec}\left(E_{N/2\times T}\right)\in R^{NT/2}$ and $e_{2}^{T}=\left[1,1\right]$. Combining (17) and (19), we obtain the equality constraints:

(22) $$\left[\begin{array}{c} \tilde{A}\\ \tilde{B} \end{array}\right]u=\left[\begin{array}{c} c\\ c \end{array}\right],$$

where $\tilde{A}=e_{T}^{T}⊗I_{N}$, and $\tilde{B}={\left(B⊙I_{N}\right)}^{T}$. The inequality constraints can be presented in the form:

(23) $$\left[\begin{array}{c} R\\ Z^{\left(1\right)}\\ Z^{\left(2\right)}\\ Z^{\left(3\right)} \end{array}\right]u\leq \left[\begin{array}{c} \hat{e}\\ {\tilde{I}}^{\left(max,1\right)}\\ {\tilde{I}}^{\left(max,2\right)}\\ {\tilde{I}}^{\left(max,3\right)} \end{array}\right],$$

where $R=I_{NT/2}⊗e_{2}^{T}$ and $Z^{\left(l\right)}=I_{T}⊗{\left(r^{\left(l\right)}\right)}^{T}$ for l∈{1,2,3}.

The objective functions can also be rewritten using Equation (18). For the linear function, we have:

(24) $$\begin{array}{c} \Psi \left(u\right)=\mathrm{vec}\left(e_{N}^{T}Uw\right)={\left(w⊗e_{N}\right)}^{T}u=d^{T}u. \end{array}$$

The other functions can be reformulated to the quadratic form:

(25) $$\begin{array}{c} \Psi \left(u\right)=\frac{1}{2}u^{T}Qu+d^{T}u+\mathrm{const}, \end{array}$$

where ***Q*** and ***d*** are given by:

- *Quadratic I:*
  (26) $$\begin{array}{c} Q=D_{w}^{2}⊗\sum_{l=1}^{3}r^{\left(l\right)}{\left(r^{\left(l\right)}\right)}^{T},\;d=-{\left[\begin{array}{c} {\underline{I}}^{\left(max,1\right)}\left(D_{w}⊗{\left(r^{\left(1\right)}\right)}^{T}\right)\\ {\underline{I}}^{\left(max,2\right)}\left(D_{w}⊗{\left(r^{\left(2\right)}\right)}^{T}\right)\\ {\underline{I}}^{\left(max,3\right)}\left(D_{w}⊗{\left(r^{\left(3\right)}\right)}^{T}\right) \end{array}\right]}^{T}e_{3}. \end{array}$$
  The derivations of ***Q*** and ***d*** in (26) are given in Appendix B.
- *Quadratic II:*
  (27) $$\begin{array}{c} Q=D_{w}e_{T}e_{T}^{T}D_{w}⊗I_{N},\;d=-{\left(e_{T}^{T}D_{w}⊗I_{N}\right)}^{T}c. \end{array}$$
- *Penalized quadratic form with smoothness constraints:*
  (28) $$\begin{array}{c} Q=D_{w}e_{T}e_{T}^{T}D_{w}⊗I_{N}+\alpha \left(L^{T}L⊗I_{N}\right),\;d=-{\left(e_{T}^{T}D_{w}⊗I_{N}\right)}^{T}c. \end{array}$$
  Appendix C contains the derivations of ***Q*** and ***d*** in (28). By setting α=0, we obtain ***Q*** and ***d*** in (27).

Considering the constraints in (22) and (23), the set of feasible regions takes the form:

(29) $$\Omega =\left\{u\in B^{NT}|\;\left[\begin{array}{c} \tilde{A}\\ \tilde{B} \end{array}\right]u=\left[\begin{array}{c} c\\ c \end{array}\right],\;\left[\begin{array}{c} R\\ Z^{\left(1\right)}\\ Z^{\left(2\right)}\\ Z^{\left(3\right)} \end{array}\right]u\leq \left[\begin{array}{c} \hat{e}\\ {\tilde{I}}^{\left(max,1\right)}\\ {\tilde{I}}^{\left(max,2\right)}\\ {\tilde{I}}^{\left(max,3\right)} \end{array}\right]\right\}$$

For objective functions (24) and (25), the scheduling problem (10) can be reformulated to the following single-vector problem:

(30) $$\begin{array}{c} \min_{u\in \Omega }\Psi \left(u\right), \end{array}$$

which can be solved with various binary optimization solvers.

### 3.2. Algorithmic Approach

We herein do not assume that matrices $\tilde{A}$ and $\tilde{B}$ in (22), and ***R*** and $Z^{\left(l\right)}$ for l∈{1,2,3} in (23) are totally unimodular because $\left\{{\tilde{I}}^{\left(max,l\right)}\right\}$ are real-value measured currents; hence, a binary relaxation to the LP problem is not justified. To solve the BLP problem, that is, problem (30) with the objective function in (24), many methods such as the cutting planes, branch-and-bound, branch-and-cut, and heuristic routines can be used. In our approach, we solve this problem using the intlinprog function from the Optimization Toolbox in MATLAB 2020b using default settings. This function is addressed to solve a mixed-integer linear programming (MILP) problem. We defined all variables as integers bounded to the range {0,1}.

Problem (30) with the other objective functions is more challenging, and there is no specific solver in MATLAB 2020b for solving the BQP problem. We studied the various algorithmic approaches described below.

#### 3.2.1. Frank—Wolfe Algorithm

The FW algorithm [4] dates back to the 1950s; however, its popularity is still noticeable in various research areas [63,64]. This algorithm is based on the concept of SLA of the objective function with a first-order Taylor expansion. The original version of the FW algorithm can also be used to solve the BQP problem under the assumption that the objective function is convex and differentiable, a set of feasible regions is convex, and a linearized version of the original problem is easy to solve.

Note that Ω in (29) is a compact convex set in $R^{NT}$ because it results from the intersection of hyperplanes and closed half-subspaces given by linear equality and inequality constraints. The objective function Ψ is at least weakly convex because it is a quadratic function with a possibly semi-positive defined matrix ***Q***. Hence, problem (30) can be solved using the following linear approximations:

(31) $$\begin{array}{c} \min_{u\in \Omega }\Psi \left(u_{k}\right)+\nabla_{u}\Psi {\left(u\right)}^{T}\left(u-u_{k}\right),\;\mathrm{for}\;k=0,1,2,\ldots \end{array}$$

To solve problem (31), we used the following Algorithm 1:
**Algorithm 1:** FW Algorithm
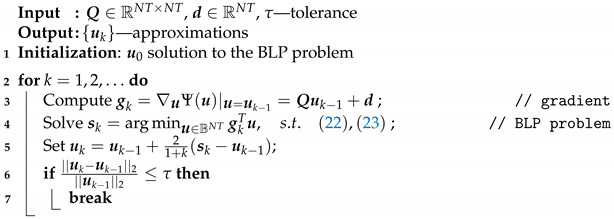


The BLP problem in line 4 of Algorithm 1 was solved using the intlinprog function from MATLAB. The gradient $g_{k}$ is Lipschitz continuous with respect to the Frobenius norm, which means that Algorithm 1 has a linear convergence with the rate O(1/k).

Algorithm 1 can be run with the negative gradient $g_{k}$, which is equivalent to the following update rule:

(32) $$\begin{array}{c} u_{k}=u_{k-1}-\eta_{k}s_{k}, \end{array}$$

where $\eta_{k}$ is the step length. Formula (32) can be regarded as the standard gradient descent update rule. In the experiments, we set $\eta_{k}=1$.

#### 3.2.2. Successive Linear Approximations

SLA [5] is based on a concept similar to that of the FA algorithm. In this approach, the BQP problem given by (30) is reformulated as follows:

(33) $$\begin{array}{c} \min_{u\in \Omega ,\zeta \in R}\zeta +d^{T}u,\;s.t.\;\frac{1}{2}u^{T}Qu\leq \zeta ,\;\zeta \geq 0. \end{array}$$

The objective function in (33) is linear, but the inequality constraints are nonlinear. However, the quadratic constraints can be linearized using the first-order Taylor expansion, similar to the FW algorithm. For the *k*-th iterative step, the inequality constraint $\frac{1}{2}u^{T}Qu\leq \zeta$ can be linearized around the point $u_{k-1}$ as follows:

(34) $$\begin{array}{ccc} \Phi (u) & = & \frac{1}{2}u^{T}Qu-\zeta ≅\frac{1}{2}u_{k-1}^{T}Qu_{k-1}+\nabla_{u}\Phi {\left(u\right)}^{T}{|}_{u=u_{k-1}}\left(u-u_{k-1}\right)-\zeta \\ & = & -\frac{1}{2}u_{k-1}^{T}Qu_{k-1}+u_{k-1}^{T}Qu-\zeta \leq 0. \end{array}$$

The inequality (34) can be expressed in the form of the following matrix inequality:

(35) $$\begin{array}{c} \left[u_{k-1}^{T}Q,\;-1\right]\left[\begin{array}{c} u\\ \zeta \end{array}\right]\leq \frac{1}{2}u_{k-1}^{T}Qu_{k-1}. \end{array}$$

Assuming $\tilde{u}=\left[\begin{array}{c} u\\ \zeta \end{array}\right]$, problem (33) for the *k*-th iterative step can be rewritten as:

(36) $$\begin{array}{c} \min_{\tilde{u}\in B^{NT+1}}{\tilde{d}}^{T}\tilde{u},\;s.t.\;\left[u_{k-1}^{T}Q,\;-1\right]\tilde{u}\leq \frac{1}{2}u_{k-1}^{T}Qu_{k-1},\;\zeta \geq 0,\;\mathrm{and}\;u\in \Omega , \end{array}$$

where $\tilde{d}=\left[\begin{array}{c} d\\ 1 \end{array}\right]$. Problem (36) is a standard BLP problem, and we solve it using the intlinprog function.

## 4. Numerical Simulations

The scheduling algorithms discussed were extensively tested using various topologies of farms and their settings, imitating real EV traffic on EV parking lots. The test cases and a description of the testing environment are presented below.

### 4.1. Setup

Without loss of generality, we assumed a 12-h time horizon from 6 p.m. to 6 a.m., divided into regular time slots of 7.5 min. Hence, T=96 in the proposed model. For an overnight charging plan, the EVs to be charged usually arrive at a parking lot in the evening. We assumed that the plug-in time for each EV can be modeled with a normal distribution with a mean of 8.30 p.m. and a standard deviation of 75 min. The earliest plug-in time was restricted to 6 p.m. The initial energy demand $c_{n}$, that is, the number of time slots required to charge the *n*-th EV to the desired SoC level is modeled with an integer uniform distribution in the range [4,30] for n=1,…,N. This indicates that the minimum charging time was 1 h, and the longest charging period did not exceed 6 h. The plug-off time was also modeled with the same distribution in the range $\left[t_{plug-in}+C_{n},\;T\right]$.

The final energy demands were determined using the correction procedure in the preprocessing stage. This procedure aims to correct the initial energy demands in a manner that guarantees the feasibility of problem (30) with a linear objective function (24). If the problem is infeasible, a subset of the EVs with the highest energy demands is selected, and their coefficients $\left\{c_{n}\right\}$ are reduced simultaneously until it is found to be feasible. If this point is obtained, then the energy demand for each EV in this subset is individually upgraded to reach the border of the feasible region. In practice, EVs with the lowest priority of charging will be selected to correct their energy demands.

We analyzed farms containing various numbers of charging stations. For the smallest farm, we assume eight two-link charging stations, which gives us 16 charging points, that is, N=16. We also tested scenarios with *N* = 32, 64, 128, and 256. Obviously, a number of ports do not have to have a multiplicity of two, but it must be an even number. If there are fewer EVs for scheduling, some ports are empty; consequently, the corresponding rows in the scheduling matrix ***U*** will have all-zero entries. For Type-II stations, the charging rate is usually limited to 16 A; however, it is not a constant parameter within a charging period. Moreover, the rates can be different for each phase line, when using three-phase chargers. Several research papers, for example, [65,66], report that the charging current of an EV Li-ion battery has almost a constant value from the plug-in (after a short starting period) to approximately half of the maximum SoC level, and then diminishes exponentially with a negative decay. Following this observation, we assume that half of the randomly selected EVs for each phase in the analyzed system charge with a maximal charging rate of 16 A, and the others have the rates determined by a uniform distribution in the range [1.6,16) A. The phases are treated independently, indicating that there could be an unbalanced load of the three-phase lines.

The proposed scheduling schemes are designed such that the maximal line current $I_{t}^{\left(max,l\right)}$ of the entire farm is not exceeded in each time slot, and this limit can vary with the time horizon. However, considering typical real charging scenarios and for simplicity of simulations, we assume that this limit is neither time nor phase dependent, that is, $I_{max}=I_{t}^{\left(max,l\right)}$ for ∀t∈T and l∈{1,2,3}. For each test case, the maximal line current was set according to the data given in Table 1. Owing to the current limit, there is a limited number of EVs that can be charged in one time slot. This number, denoted by $L_{x}$, is also given in Table 1 for a constant charging rate of 16 A from each line.

In this study, we proposed various binary algorithmic approaches to solve the scheduling problem in (30). The results are presented in the following cases.

- BLP: Binary linear programming (BLP) with objective function in (24);
- Q1-FW: Binary quadratic programming (*Quadratic I*), with the objective function expressed by (25) and (26) and solved with the Frank–Wolfe (FW) algorithm;
- SmQ2-FW: Binary quadratic programming (*Quadratic II*), with the objective function expressed by (25) and (28) (including the smoothness), and solved with the FW algorithm;
- SmQ2-NG-FW: Binary quadratic programming (*Quadratic II*), with the objective function expressed by (25) and (28) (including the smoothness), and solved with the negative gradient FW algorithm—rule (32);
- SmSLA: Binary quadratic programming (*Quadratic II*), with the objective function expressed by (25) and (28) (including the smoothness), and solved with the successive linear approximations (SLA);
- FA-FS: First-arrive-first-serve (FA-FS) approach.

Q1-FW is closely related to the scheduling problem given in [38] owing to the formulation of the objective function. However, our two-link constraints are quite specific, and to the best of our knowledge, there is no competitive algorithmic strategy for a simple comparison. FA-FS is a heuristic strategy that turns on charging each EV as quickly as possible. If two EVs are simultaneously plugged into one charging station, their selection is random. This strategy yields the fastest charging; however, it does not have any admissible power constraints.

The algorithms were implemented in MATLAB 2020b and run on a machine supplied with a 4-core Intel Core-i7 CPU, 32-GB RAM, and an SSD drive.

### 4.2. Results

**Figure 2:**
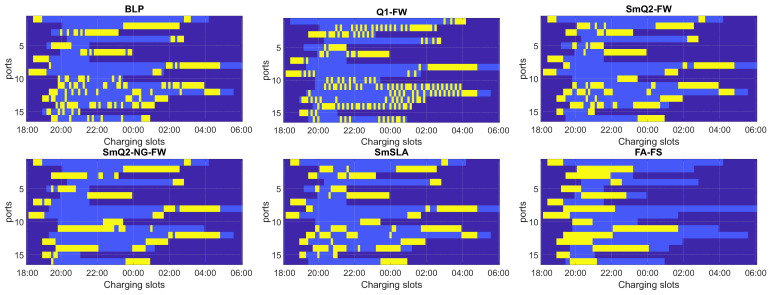
One MC run of charging schedules obtained with the tested algorithms for N=16 and random charging rates. Charging slots are in yellow, available time slots are in light blue, and unavailable time slots are in dark blue.

**Figure 3:**
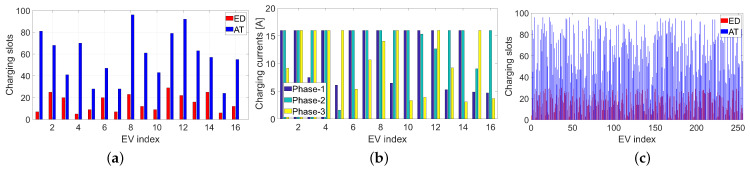
Charging settings: (**a**) ED and AT parameters for N=16 and random charging rates; (**b**) charging rates for N=16; (**c**) ED and AT parameters for N=256 and a constant charging rate.

**Figure 4:**
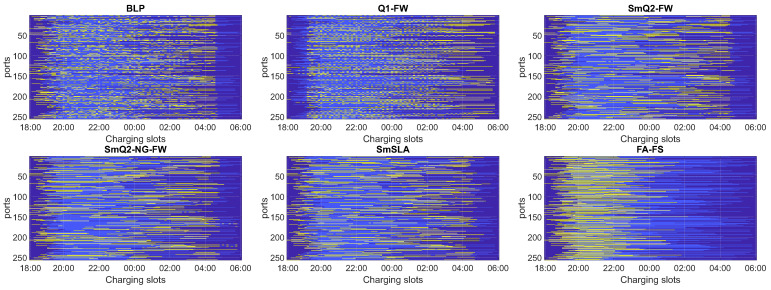
One MC run of charging schedules obtained with the tested algorithms for N=256 and a constant charging rate.

**Figure 5:**
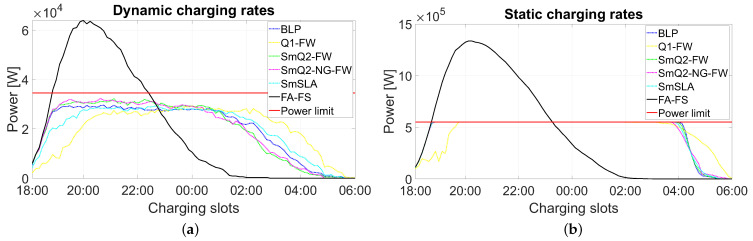
Averaged total power consumed by the whole farm versus the time slots: (**a**) small farm with N=16 and random charging rates; (**b**) large farm with N=256 and a constant charging rate.

**Figure 6:**
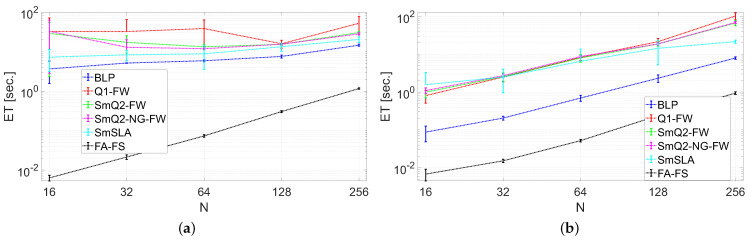
Averaged elapsed time (ET) for running the schedulers with (**a**) random charging rates and (**b**) a constant charging rate.

To statistically validate the algorithms, Monte Carlo (MC) analysis was performed using 30 runs for each algorithm. In each snapshot, the charging rates, energy demands, plug-in, and plug-off times were generated randomly according to the procedures discussed above. The selected single-run results are shown in Figure 2, Figure 3 and Figure 4, while the MC statistics are presented in Table 2, Table 3 and Table 4, and in Figure 5 and Figure 6.

The algorithms were validated using various criteria and datasets. Figure 2 presents a graphical visualization of the charging matrices ***U*** obtained in one selected MC run with the tested algorithms for the smallest farm containing 16 ports (N=16) and random charging rates. The vertical axis corresponds to the ports, and the horizontal axis represents the time slots. The yellow fields correspond to the switch-on state. The light blue fields show the time slots between the plug-in and plug-off times for each EV (each row of the matrix). The dark blue fields denote unavailable time slots. The distributions of energy demand (ED) after using the correction procedure and available time (AT), which were used to obtain the results in Figure 2, are illustrated in Figure 3a in the form of time slot bars. Both ED and AT are expressed in terms of the number of time slots. The charging rates in this case are shown in Figure 3b, separately for each phase.

Similar charging schedules are shown in Figure 4 for the largest analyzed farm. For this case, we set N=256 and a constant charging rate of 16 A for each phase line. The distributions of the ED and AT parameters are shown in Figure 3c. Note that a small number of EVs had no assigned ED. This results from using the correction algorithm to guarantee feasibility.

The algorithms were also quantitatively validated using various metrics, which are listed in Table 2, Table 3 and Table 4. The results obtained for the smallest farm with N=16 and random charging rates are listed in Table 2. Table 3 and Table 4 contain the results obtained for the largest farm with constant and random charging rates, respectively.

One of the most important criteria for validating the correctness of the algorithm is to check if the constraints are satisfied. The equality constraints for the EDs were validated with the residual error $r_{c}=||c-Ue_{T}{\left|\right|}_{2}$. Table 2, Table 3 and Table 4 contain both the mean and median values of the residual error $r_{c}$ obtained from the MC runs.

We also verified that max{SU}=1 for each proposed algorithm and for each analyzed scenario. This observation leads to the conclusion that each algorithm satisfies the two-link constraint in (9), which is necessary to guarantee that only one port in each charging station is active in one time slot.

Another criterion is the smoothness measure expressed by the function $F_{smooth}=u^{T}Q_{L}u$, where u=vec(U) and $Q_{L}=L^{T}L⊗I_{N}$ is given in (28). The mean values and standard deviations (in parentheses) of $F_{smooth}$ are also listed in the tables. Parameter $S_{x}$ denotes the total number of switching-on operations. The lower bound of this parameter was *N*. The tardiness, expressed by the number of time slots wherein at least one charging station is switched on, is referred to as $T_{x}$. Parameter $L_{x}$ denotes the maximal number of EVs charged in any time slot. The percentage of MC runs wherein all EVs are charged up to their desired SoC level over the entire time horizon is expressed by $L_{f}$. If $L_{f}=0$, it means that in each MC run, at least one EV is not charged according to the desired level. All parameters $S_{x}$, $T_{x}$, $L_{x}$, and $L_{f}$ are expressed as median values.

The total power consumed by the entire farm versus time slots is illustrated in Figure 5a for the smallest farm with N=16 and random charging rates, and in Figure 5b for the largest farm with N=256 and a constant charging rate. The power in the *t*-th time slot is calculated as $P_{t}^{\left(max\right)}=U_{s}\sum_{n=1}^{N}u_{nt}\sum_{l=1}^{3}r_{n}^{\left(l\right)}$, where $U_{s}=230\;V$ is the phase voltage. The red horizontal line determines the maximal load owing to the limit of $I_{max}$.

The computational complexity of the proposed algorithmic strategies was evaluated in terms of the elapsed time (ET) in seconds. The plots of the averaged ET for running the proposed algorithms on the scheduling problem in the farm with N∈{16,32,64,128,256} are shown in Figure 6a for random charging rates and in Figure 6b for a constant charging rate. The whiskers determined the standard deviation.

### 4.3. Discussion

Experiments were conducted for a variety of scheduling problems. Figure 3a,c show that ED and AT parameters are highly diversified in a wide range of their possible values in our tests. This reflects the real charging scenarios. Moreover, the charging rates considerably change with time and phase lines. In practice, the charging currents can be different for each phase line in the same time slot. This case was considered in our simulations, as shown in Figure 3b.

The experiments demonstrated that the most challenging scheduling problems, due to computational issues, were scenarios with random charging rates. Such problems were more difficult to tackle even for a small-scale farm (N=16) than for a large-scale farm (for N=256) but with a constant charging rate. The residual errors and the $L_{f}$ measure presented in Table 3 and Table 4 clearly confirm this statement. This observation is theoretically justified as the feasible region determined by the inequality constraints in (23) has the form of a complex polytop, with more vertices when $r_{n}^{\left(l\right)}$ varies with *n* and *l*. Our experiments showed that for a practical case with varying charging rates, we have a robust algorithm for scheduling problems, even when the number of EVs is large. It is the SmSLA strategy, which always (for each MC run) yields the solution fully satisfying all constraints, that is, the mean $r_{c}$ is marginally small and $L_{f}=100\%$. However, this algorithm wins only with respect to this criterion, and it is not the best one in each analyzed competition.

Regarding all the criteria used for validating the algorithm, one can conclude that there is no candidate that wins in all tested categories. The FA-FS provides the results with the shortest tardiness (lowest $T_{x}$ in the tables) and the shortest ET (see Figure 6). Unfortunately, it does not have any embedded power constraints. This leads to a strong overload effect, as clearly shown in Figure 5. Hence, this algorithm is not acceptable for solving practical scheduling problems. FA-FS was used in our experiments only to demonstrate the load peak effect. All other algorithms prevent the overload effect and provide uniform energy consumption over a wide time window, as depicted in Figure 5 for each analyzed scenario. For a constant charging rate, BLP, SmQ2-FW, SmQ2-NG-FW, and SmSLA behaved similarly, and only Q1-FW provided a slightly delayed energy consumption profile. In this scenario, the farm works with the maximum acceptable power in a wide time window (from approximately 6.30 p.m. to 4 a.m.). When the charging rates are not constant, the farm works with a power slightly below the limit (the red line in Figure 5), which is still acceptable. Moreover, the energy consumption is approximately uniform from 7 p.m. to 1 a.m. for nearly all algorithms. Subsequently, a decreasing trend in energy consumption was observed until 6 a.m.

With regard to tardiness ($T_{x}$), Q1-FW and SmSLA are the least competitive. However, this criterion is correlated with the $L_{x}$ measure, and it is thus obvious that fast charging is related to higher energy consumption. Q1-FW and SmSLA have a lower number of EVs in the most energy-consuming period. Hence, their schedules are safer with respect to energy consumption and maintain a higher margin to the power limit.

Another crucial criterion is the smoothness of the charging profile for each EV, which is related to the number of switching on/off operations at each charging station. The results presented in the tables demonstrate that SmQ2-NG-FW provides the smoothest charging profiles with a relatively low number of switching operations (parameter $S_{x}$). The worst results in this category were obtained by the BLP and Q1-FW. This observation can be justified by the fact that both BLP and Q1-FW do not involve any procedures for enforcing smoothness, whereas the others (excluding FA-FS) are based on the BQP strategy, where the quadratic term contains the smoothness penalty. Moreover, the quadratic II approach seems to be more favorable with respect to smoothness than the quadratic I approach. Furthermore, the results listed in the tables can be confirmed by the single-run charging matrices presented in Figure 2 and Figure 4. The charging profiles of SmQ2-NG-FW are the smoothest, but SmQ2-FW and SmSLA are only slightly worse, which is in favor of the latter considering all the criteria.

Figure 2 and Figure 4 also show that SmQ2-FW, SmQ2-NG-FW, and SmSLA better satisfy the rule of early charging than BLP and Q1-FW. Obviously, FA-FS is the best in this category; however, it is disqualified due to the power limit criterion (as discussed above). This rule is enforced by the matrix $D_{w}$ in (12), (13), and (16), and it aims to enforce charging as early as possible. Obviously, the similar rule is incorporated in (11) via vector ***w*** but BLP seems to tackle worse in this aspect, as demonstrated in Figure 2 and Figure 4.

The algorithms were evaluated with respect to the computational complexity expressed by the ET criterion. In this category, BLP is substantially faster than the others (excluding FA-FS) when a constant charging rate is applied (see Figure 6). For the random charging rates, the difference in ET was not very large. Interestingly, an increase in the number of EVs scales linearly for BLP, and there is no simple relation between ET and *N* for Q1-FW, SmQ2-FW, and SmQ2-NG-FW. This is probably owing to the high computational cost of processing a much more complicated polytop determined by (23) when random charging rates are used. For N=256, Q1-FW, SmQ2-FW, SmQ2-NG-FW, and SmSLA have approximately similar ET values for random and constant charging rates. BLP is much faster for each scenario and provides satisfactory results for meeting the constraints. Hence, it can be used in the precomputing stage to check if a feasible solution exists, given the input data.

### 4.4. Engineering Aspects

The above experiments allowed us to select SmSLA as the most optimal algorithm for the scheduler. The charging matrix ***U***, together with the real-time intervals of the time slots, is passed to the CCU where real-time decisions on the current charging status of EVSEs are taken for each time slot. If $u_{nt}=1$, the *n*-th charging point is switched on in the *t*th time slot. Otherwise, it is switched off. The scheduler is run each time a new EV is connected or disconnected to/from an EVSE, the scheduling period is over, or the CCU detects a considerable change in the charging currents. When a scheduler is called, the initialization procedures (which are not described herein) determine the current state of charging for each active EV. The EDs were updated using the previous charging matrices {U}. For a new EV, the ED is evaluated using its individual charging curve, user demands, and historical charging data.

The charging problem may not have any solution, given real input data. This is a normal situation, not resulting from an algorithmic issue but rather due to a lack of a feasible solution to a given problem. To tackle this problem, BLP is used in the initialization procedure to detect this case, and if it occurs, the correction procedure is applied to decrease the EDs for the selected group of already charging EVs or for a new EV. This procedure was governed by the ED correction procedure.

## 5. Conclusions

In this study, we proposed a new model for on–off scheduling of EV charging, assuming that each three-phase charger is equipped in two ports that can be alternately served. The scheduler considers individual charging rates and maximal currents that supply the entire farm separately for each phase. For this model, we analyzed various binary algorithm approaches. All algorithms were validated with respect to multiple criteria, including constraint satisfying conditions, energy limit, tardiness, and charging profile smoothness. The experiments demonstrated that only SmSLA can yield the correct solution that satisfies all constraints for each MC run of each testing scenario. It also ensures smooth charging profiles. Unfortunately, this algorithm has a relatively long tardiness and is not the fastest. BLP is much faster than the others; however, it does not enforce smoothness. However, BLP could be a good choice for the preprocessing stage to guarantee feasibility.

The presented results, despite being completed, suggest that the issue of the algorithmic approach is still open, and further research in this area will be performed in the future. We still admit the possibility of designing a more robust algorithm than SmSLA, which would provide smoother charging profiles and lower tardiness. Furthermore, the topic of fast checking the feasibility (without running the scheduler) would be very interesting, with a high potential for practical applications.

In summary, we proposed a new model for the on–off scheduling of two-link EV chargers and experimentally evaluated the effectiveness of various binary algorithmic approaches with regard to multiple criteria. We statistically demonstrated that the best choice is the computational algorithm based on SLA with smoothness constraints (SmSLA). It satisfies the most important criteria and constraints in all statistical tests performed.

## Figures and Tables

**Table 1:** Power limit settings: $I_{max}$—the maximal line current (in Amperes) of the whole farm, $L_{x}$—the maximal number of EVs charging in one time slot given a constant charging rate of 16 A.

	N=16	N=32	N=64	N=128	N=256
$I_{max}$	50	100	200	400	800
$L_{x}$	3	6	12	25	50

**Table 2:** Results obtained for the smallest farm with 16 ports, 16 EVs, and random charging rates. The maximal number of EVs that can be charged in one time slot is restricted to five owing to the power limit.

Algorithm	Mean ($r_{c}$)	Median ($r_{c}$)	$F_{\mathrm{smooth}}$	$S_{x}$	$T_{x}$	$L_{x}$	$L_{f}$ [%]
BLP	0.088	$1.98\times 10^{-14}$	187.7 (37.7)	107	76	4	96.7
Q1-FW	1.09	$2.54\times 10^{-14}$	191.7 (72)	105	85	4	76.7
SmQ2-FW	3.07	3.61	82.27 (38.8)	39	71	5	43.3
SmQ2-NG-FW	2.54	$2.67\times 10^{-14}$	71.33 (28.58)	41	72	5	51
SmSLA	$9.68\times 10^{-14}$	$2.53\times 10^{-14}$	100.38 (60.57)	92	81	4	100
FA-FS	0	0	31.13 (1.45)	15	53	8	100

**Table 3:** Results obtained for the largest farm with 256 ports, 252 EVs, and constant charging rate. The maximal number of EVs that can be charged in one time slot is restricted to 50 owing to the power limit.

Algorithm	Mean ($r_{c}$)	Median ($r_{c}$)	$F_{\mathrm{smooth}}$	$S_{x}$	$T_{x}$	$L_{x}$	$L_{f}$ [%]
BLP	$1.17\times 10^{-13}$	$8.05\times 10^{-14}$	3505 (238)	1909	84	50	100
Q1-FW	$1.48\times 10^{-13}$	$9.12\times 10^{-14}$	4259 (416)	2152	94	50	100
SmQ2-FW	$9.33\times 10^{-14}$	$8.73\times 10^{-14}$	924.6 (27.68)	644	87	50	100
SmQ2-NG-FW	$1.1\times 10^{-13}$	$9.09\times 10^{-14}$	772.9 (18.44)	515	93	50	100
SmSLA	$2.15\times 10^{-13}$	$1.48\times 10^{-13}$	892.6 (22.66)	621	90	50	100
FA-FS	0	0	502.3 (3.57)	242	66	123	100

**Table 4:** Results obtained for the largest farm with 256 ports, 196 EVs, and random charging rates. The maximal number of EVs that can be charged in one time slot is restricted to 80 owing to the power limit.

Algorithm	Mean ($r_{c}$)	Median ($r_{c}$)	$F_{\mathrm{smooth}}$	$S_{x}$	$T_{x}$	$L_{x}$	$L_{f}$ [%]
BLP	2.97	2.92	848.4 (240.6)	395	65	78	0
Q1-FW	0.141	$5.3\times 10^{-14}$	1322 (178)	745	94	61	90
SmQ2-FW	2.61	2.65	614 (34.17)	304	65	70	0
SmQ2-NG-FW	2.48	2.44	557 (25.4)	268	65	72	0
SmSLA	$6.29\times 10^{-14}$	$5.74\times 10^{-14}$	454.5 (38.1)	335	92	62	100
FA-FS	0	0	394.6 (12.15)	188	65	105	100

## Data Availability

The data presented in this study and the Matlab code of the discussed algorithms are available from https://github.com/RafalZdunek/On-off-scheduler.git.

## Appendix A

Let $U=\left[u_{1},\ldots ,u_{T}\right]\in B^{N\times T}$, $B=\left[\begin{array}{c} {\underline{b}}_{1}\\ \vdots \\ {\underline{b}}_{T} \end{array}\right]\in B^{T\times N}$, and $I_{T}=\left[i_{1},\ldots ,i_{T}\right]\in R^{T\times T}$ be the identity matrix. Formula (4) can be reformulated as follows:

(A1) $$\begin{array}{ccc} c & = & \mathrm{diag}\left\{UB\right\}=\left[\begin{array}{ccc} {\underline{b}}_{1}u_{1} & \; & 0\\ & \ddots & \\ 0 & \; & {\underline{b}}_{T}u_{T} \end{array}\right]=\left[\begin{array}{ccc} {\underline{b}}_{1} & \; & 0\\ & \ddots & \\ 0 & \; & {\underline{b}}_{T} \end{array}\right]\left[\begin{array}{c} u_{1}\\ \vdots \\ u_{T} \end{array}\right]\\ & = & {\left[\begin{array}{ccc} {\underline{b}}_{1}^{T} & \; & 0\\ & \ddots & \\ 0 & \; & {\underline{b}}_{T}^{T} \end{array}\right]}^{T}\left[\begin{array}{c} u_{1}\\ \vdots \\ u_{T} \end{array}\right]={\left[{\underline{b}}_{1}^{T}⊗i_{1},\cdots ,{\underline{b}}_{T}^{T}⊗i_{T}\right]}^{T}\left[\begin{array}{c} u_{1}\\ \vdots \\ u_{T} \end{array}\right]\\ & = & {\left(B^{T}⊙I_{T}\right)}^{T}\mathrm{vec}\left(U\right). \end{array}$$

## Appendix B

Function (12) can be reformulated as follows:

(A2) $$\begin{array}{ccc} \Psi & = & \frac{1}{2}\sum_{l=1}^{3}{∥{\underline{I}}^{\left(max,l\right)}-{\left(r^{\left(l\right)}\right)}^{T}UD_{w}∥}_{2}^{2}=\frac{1}{2}\sum_{l=1}^{3}{∥{\underline{I}}^{\left(max,l\right)}-\left(D_{w}⊗{\left(r^{\left(l\right)}\right)}^{T}\right)u∥}_{2}^{2}\\ \; & = & \frac{1}{2}\sum_{l=1}^{3}\left[u^{T}{\left(D_{w}⊗{\left(r^{\left(l\right)}\right)}^{T}\right)}^{T}\left(D_{w}⊗{\left(r^{\left(l\right)}\right)}^{T}\right)u-{\underline{I}}^{\left(max,l\right)}\left(D_{w}⊗{\left(r^{\left(l\right)}\right)}^{T}\right)u\right]+\mathrm{const}\\ \; & = & \frac{1}{2}u^{T}\left(D_{w}^{2}⊗\sum_{l=1}^{3}r^{\left(l\right)}{\left(r^{\left(l\right)}\right)}^{T}\right)u-e_{3}^{T}{\left[\begin{array}{c} {\underline{I}}^{\left(max,1\right)}\left(D_{w}⊗{\left(r^{\left(1\right)}\right)}^{T}\right)\\ {\underline{I}}^{\left(max,2\right)}\left(D_{w}⊗{\left(r^{\left(2\right)}\right)}^{T}\right)\\ {\underline{I}}^{\left(max,3\right)}\left(D_{w}⊗{\left(r^{\left(3\right)}\right)}^{T}\right) \end{array}\right]}^{T}u+\mathrm{const}\\ \; & = & \frac{1}{2}u^{T}Qu+d^{T}u+\mathrm{const}. \end{array}$$

## Appendix C

Function (16) can be presented in the following form:

(A3) $$\begin{array}{ccc} \Psi & = & \frac{1}{2}{∥c-UD_{w}e_{T}∥}_{2}^{2}+\frac{\alpha }{2}{∥UL^{T}∥}_{F}^{2}\\ \; & = & \frac{1}{2}{∥c-\left(e_{T}^{T}D_{w}⊗I_{N}\right)u∥}_{2}^{2}+\frac{\alpha }{2}{∥\left(L⊗I_{N}\right)u∥}_{F}^{2}\\ \; & = & \frac{1}{2}u^{T}{\left(e_{T}^{T}D_{w}⊗I_{N}\right)}^{T}\left(e_{T}^{T}D_{w}⊗I_{N}\right)u-c^{T}\left(e_{T}^{T}D_{w}⊗I_{N}\right)u\\ \; & + & \frac{1}{2}u^{T}{\left(L⊗I_{N}\right)}^{T}\left(L⊗I_{N}\right)u+\mathrm{const}\\ \; & = & \frac{1}{2}u^{T}\left(D_{w}e_{T}e_{T}^{T}D_{w}⊗I_{N}+\alpha \left(L^{T}L⊗I_{N}\right)\right)u-c^{T}\left(e_{T}^{T}D_{w}⊗I_{N}\right)u+\mathrm{const}\\ \; & = & \frac{1}{2}u^{T}Qu+d^{T}u+\mathrm{const}. \end{array}$$
